# Improving the scaling normalization for high-density oligonucleotide GeneChip expression microarrays

**DOI:** 10.1186/1471-2105-5-103

**Published:** 2004-07-29

**Authors:** Chao Lu

**Affiliations:** 1 Microarray Facility, The Centre for Applied Genomics, The Hospital for Sick Children, 555 University Avenue, Elm Wing Room 10104, Toronto, Ontario M5G 1X8, Canada

**Keywords:** Microarray, normalization, gene expression, DNA, RNA, oligonucleotide, GeneChip, scaling

## Abstract

**Background:**

Normalization is an important step for microarray data analysis to minimize biological and technical variations. Choosing a suitable approach can be critical. The default method in GeneChip expression microarray uses a constant factor, the scaling factor (*SF*), for every gene on an array. The *SF *is obtained from a trimmed average signal of the array after excluding the 2% of the probe sets with the highest and the lowest values.

**Results:**

Among the 76 U34A GeneChip experiments, the total signals on each array showed 25.8% variations in terms of the coefficient of variation, although all microarrays were hybridized with the same amount of biotin-labeled cRNA. The 2% of the probe sets with the highest signals that were normally excluded from *SF *calculation accounted for 34% to 54% of the total signals (40.7% ± 4.4%, mean ± sd). In comparison with normalization factors obtained from the median signal or from the mean of the log transformed signal, *SF *showed the greatest variation. The normalization factors obtained from log transformed signals showed least variation.

**Conclusions:**

Eliminating 40% of the signal data during *SF *calculation failed to show any benefit. Normalization factors obtained with log transformed signals performed the best. Thus, it is suggested to use the mean of the logarithm transformed data for normalization, rather than the arithmetic mean of signals in GeneChip gene expression microarrays.

## Background

The high-density oligonucleotide microarray, also known as GeneChip^®^, made by Affymetrix Inc (Santa Clara, CA), has been widely used in both academic institutions and industrial companies, and is considered as the "standard" of gene expression microarrays among several platforms. A single GeneChip^® ^can hold more than 50,000 probe sets for every gene in human genome. A probe set is a collection of probe pairs that interrogates the same sequence, or set of sequences, and typically contains 11 probe pairs of 25-mer oligonucleotides [1-3]. Each pair contains the complementary sequence to the gene of interest, the so-called perfect match (PM), and a specificity control, called the Mismatch (MM) [3]. Gene expression level is obtained from the calculation of hybridization intensity to the probe pairs and is referred to as the "signal" [4-10]. The normalization method used in GeneChip software is called scaling and is defined as an adjustment of the average signal value of all arrays to a common value, the target signal value in order to make the data from multiple arrays comparable [4,11].

The purpose of data normalization is to minimize the effects of experimental and/or technical variations so that meaningful biological comparisons can be made and true biological changes can be found among multiple experiments. Several approaches have been proposed and shown to be effective and beneficial. They were mostly from studies on two-color spotted microarrays [12-19]. Some authors proposed normalization of the hybridization intensities, while others preferred to normalize the intensity ratios. Some used global, linear methods, while others used local, non-linear methods. Some suggested using the spike-in controls, or house-keeping genes, or invariant genes, while others preferred all the genes on the array. For GeneChip data, some have proposed different models to normalize signal values or normalize probe pair values [10,20-24]. Despite the presence of other alternatives, many biologists still use the default scaling method and consider that such method is satisfactory and is useful to identify biological alterations [23,25,26]. With the increasing awareness and usage of GeneChip technology and willingness to continue to use GeneChip software among many biologists, it is worth improving the performance or correcting the problems of the software. In this report, the author has demonstrated that in the scaling algorithm excluding 2% of the probe sets with the highest and the lowest values did not have much benefit. However, the logarithmic transformation of signal values prior to scaling proved to be the optimum normalization strategy and is strongly recommended.

## Results

The statistical algorithm in current GeneChip software (MAS 5 and GCOS 1) for gene expression microarray data has eliminated the negative gene expression values, a problem present in earlier versions of the software [5,7]. It uses a robust averaging method based on the Tukey biweight function to calculate the gene expression level from the logarithm transformed hybridization data [3-5,11]. The reported data of a probe set is the antilog of the Tukey biweight mean multiplied by a *SF *and/or a normalization factor (*NF*_*affy*_). When both the *SF *and *NF*_*affy *_are equal to 1, there is no normalization or manipulation of original data. Both *NF*_*affy *_and *SF *are computed in virtually the same way. *NF*_*affy *_is calculated in comparison analysis to compare the array average of one experiment with that of a baseline experiment, while *SF *is obtained from the signal average of one experiment comparing with a common value, the target signal in absolute analysis [3-5,11,22]. The average value used in GeneChip is a trimmed average. It is not calculated from all probe sets, but from 96% of the probe sets after the 2% of the probe sets with the highest and the 2% of the lowest signals were removed.

In this report, a total of 76 experiments with rat U34A GeneChip were analyzed. As shown in Table 1, the total hybridization signals varied although all arrays were hybridized with the same amount of biotin-labeled cRNA and scanned with the same scanner of identical settings. The array of the highest hybridization intensities had 2.8 times more signals than that of the lowest. The average array signals had 25.8% variation in terms of coefficient of variation. The mean signals were significantly greater than the median signals on each array, indicating a non-normal distribution. The density plot showed a long-tailed and skewed distribution (not shown) and the average of such data is known to be sensitive to the larger values in the data set.

The rat U34A GeneChip contained 8799 probe sets; hence 2% was about 176 probe sets. The sum of the 2% of the probe sets with the lowest signals accounts for less than 0.1% of the total signals (0.05% ± 0.01%, mean ± SD, n = 76) and its impact on *SF *calculation can be ignored. However, the sum of the 2% of the probe sets with the highest signals, the *TrimTotal *as used in this report, was responsible for about 40% of the total signals (from 34% to 54%, Table 1). The remaining 96% of the probe sets used for *SF *calculation, produced only about 60% of the signals. Excluding 4% of the probe sets did not reduce the variation, but rather slightly increased the variation, which in turn resulted in a wider range of *SF*s (Table 1). It was also found that the *TrimTotal *was highly correlated with total signal (R = 0.928), but less with medians (R = 0.536) and the mean of log signals (R = 0.643). The trimmed percentage (*Tp*) was found to be negatively associated with the median (R = 0.558, b = -1.116) and the mean of log signals (R = 0.495, b = -0.968), but not with the total signal of all probe sets.

**Figure 1:**
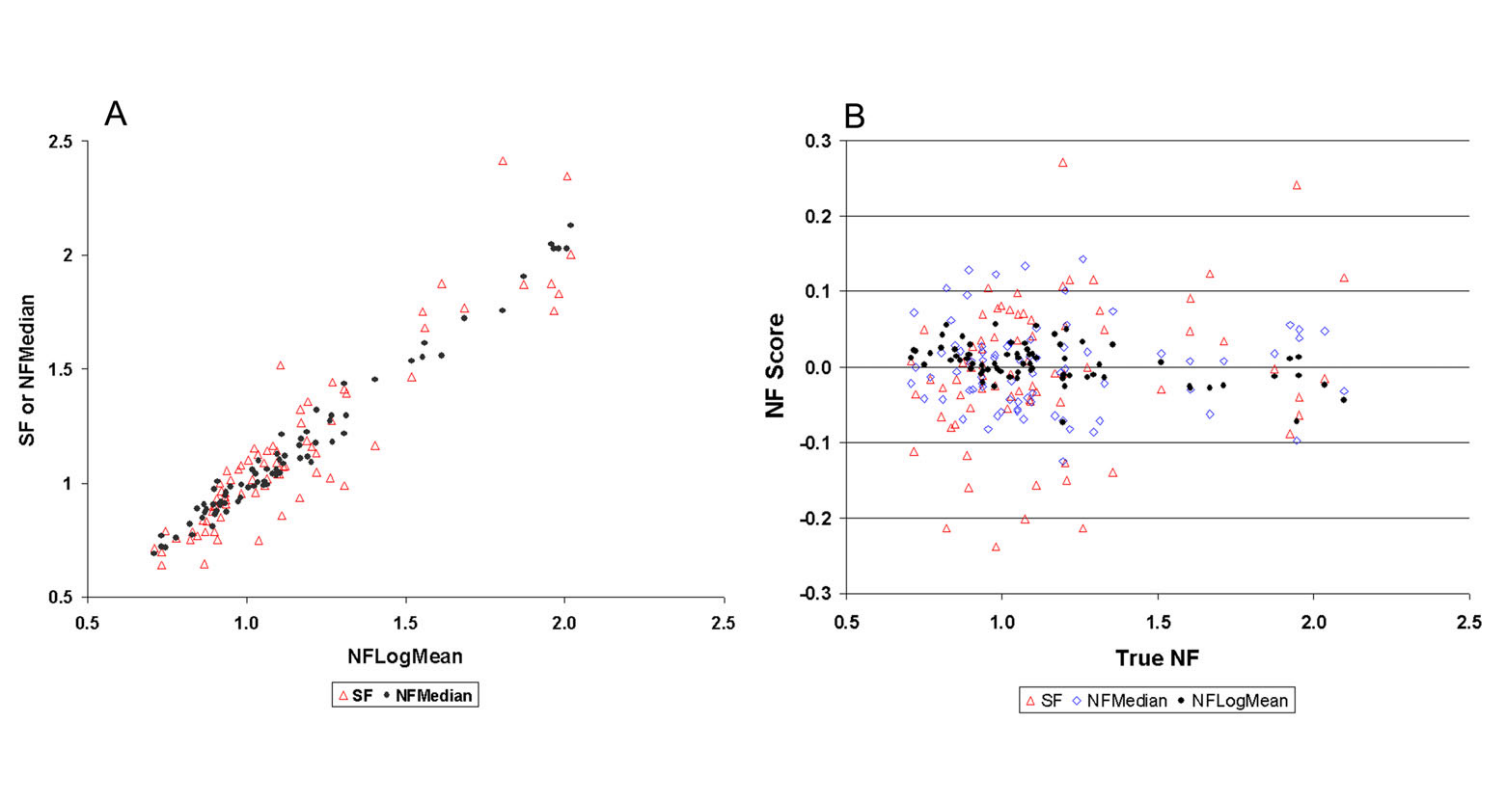
(A) Comparison among different normalization factors. *NFLogMean *(x-axis) is plotted against *SF *(red open triangle) and *NFMedian *(black closed circle). The correlation between *NFLogMean *and *NFMedian *is higher (R = 0.971) than that between *NFLogMean *and *SF *(R = 0.918). (B) The NF score, *NFscore*, for *SF *(red open triangle), *NFMedian *(blue open diamond) and *NFLogMean *(black closed circle) is expressed as a function of respective '*true NF*'. *NFTrimLogMean *is not shown here to simplify the graph since it is similar to *NFLogMean*. See also in* Methods*.

Among other approaches to global linear normalization, one can also use the median signal or the mean of logarithm transformed signals to calculate the NF. *NFLogMean *showed a higher correlation with *NFMedian *than with *SF*. There were larger differences between *NFLogMean *and *SF *than those between *NFLogMean *and *NFMedian *(Fig. 1). To test if the larger difference was a result of removing 4% of the probe sets from the calculation, another NF, the *NFTrimLogMean *was obtained using the same data as for *SF*, but with a log transformation. There is a very significant correlation between *NFTrimLogMean *and *NFLogMean *(R = 0.9998). The 4% of the probe sets that was removed from *NFTrimLogMean *calculation reduced the total data by only 4% after log transformation.

Since it is impossible to obtain the true normalization factor, an average of the four global linear *NF*s mentioned above was used instead to estimate the 'true' NF. To compare them with the true NF, a score (*NFscore*) is introduced. Each NF is calculated against the respective 'true' NF to obtain its *NFscore*. The average *NFscore *(± SD) is 7.01% (± 6.24%), 4.51% (± 3.48%), 2.25%(± 2.33%) and 1.95% (± 1.61%), and the sum of *NFscore *is 5.33, 3.43, 1.71 and 1.48 for *SF*, *NFMedian*, *NFTrimLogMean *and *NFLogMean*, respectively (Fig. 1). The sum of *NFscore *indicated an accumulated variation from the true NF, and the larger the number, the larger the accumulated variation. An attempt to add a 5th NF obtained from the arithmetic mean of all probe sets of the array was also made to calculate and compare *NFscore *with each NFs, and the results showed the same conclusion (data not shown). It is fair to conclude that *NFLogMean *produced the least variation.

## Discussion

Logarithmic transformation is a well-accepted approach for stabilizing variance and has become a common choice for data transformation and normalization for spotted microarrays [12,16]. Much improvement has been made in GeneChip microarray technology and accompanying software during the past few years. The current version of GeneChip software has improved its performance and is better than the earlier versions that used the Average Difference to express levels of gene expression [3,4]. However, the normalization algorithm was inherited and remains the only and default option for gene expression data processing in both MAS 5 and the newly released GeneChip Operating Software (GCOS) software. They continue to use the arithmetic mean of signals to obtain the *SF *in absolute analysis (single array) and the *NF *in comparison analysis (two arrays) [3-5,7,11,22]. It is clearly shown here that the trimmed average and the resulting *SF *had a larger variance than the median-based NF, or the NF based on the mean of log transformed signals. Similar results were observed in other GeneChip expression arrays, such as mouse U74A and human U133A (data not shown). Elimination of the highest and the lowest 2% of the probe set signals did not stabilize the trimmed means. When intra-array variance was reduced by 40%, this approach cannot be considered to be optimal. The logarithmic transformation of signals stabilized the variation well and made the normalization process much less dependent upon the mean and less affected by the outliers.

Although simple and popular, the global linear normalization has its drawbacks, especially when the relationship among multiple experiments or genes is not linear. To address such problems, several methods have been proposed to conduct local and non-linear normalization, [12,14-17,20,22,27]. Data normalization is a very critical and important step for microarray data mining process. The use of different approaches to normalization may have a profound impact on the selection of differentially expressed genes and conclusions about the underlying biological processes especially when subtle biological changes are investigated [12,16,28].

## Conclusions

Normalization of microarray data allows direct comparison of gene expression levels among experiments. A global linear normalization, called scaling has been widely used in GeneChip microarray technology for gene expression analysis. The scaling factor (*SF*) is calculated from a trimmed average of gene expression level after excluding the 2% of the data points of the highest values and the lowest values. It is shown here that the 2% of the probe sets of the highest signals contained from 34% to 54% of the total signals. Elimination of the outliers did not reduce, but increased the variation among multiple arrays. Instead, normalization factors obtained from the mean of the log transformed signals had the best performance. Thus, the current scaling method, although widely used, is not optimal and needs further improvement. The mean of logarithm transformed signals is highly recommended to use for normalization factor calculation.

## Methods

### GeneChip experiments and data

Total RNA was isolated from rat tissues or cells in Trizol reagent and purified with Qiagen Rneasy kit. cDNA was synthesized in presence of oligo(dT)24-T4 (Genset Corp, La Jolla, CA) and biotinlated UTP and CTP were used to generate biotin labeled cRNA according to the recommended protocols [29]. Rat genome microarray, U34A GeneChip (Affymetrix Inc., Santa Clara, CA) was used and hybridized with 15 μg of gel-verified fragmented cRNA. Hybridization intensity was scanned in GeneArray 2500 scanner (Agilent, Palo Alto, CA) with Microarray Suite (MAS) 5.0 software [4]. Data from a total of 76 independent GeneChip experiments were used in this study.

### Normalization factor (NF)

Gene expression data exported from MAS 5.0 were submitted to a Perl script to calculate different normalization factors. In the scaling approach, a trimmed average signal is calculated after excluding 2% probe sets with the highest signals and 2% with the lowest signal values. The scaling factor (*SF*) is obtained using equation (1) in comparison with a chosen fixed number, called the target signal (*TS*) and is verified with the results from MAS 5.0 of the same settings [3,4,11].

*SF*_*j *_= *TS */ *S*_*TrimMeanj *_    (1)

Other normalization factors for comparison were obtained by the following:

*NFMedian*_*j *_= *TS */ *S*_med*j *_    (2)

*NFLogMean*_*j *_= 2 ^*nf*^_*j*_


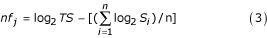


where *i *= 1..., n represents the probe sets, *j *= 1..., J represented the array experiments, *Si *is the signal of the anti-log of a robust average (Tukey biweight) of log(PM-MM) reported from MAS 5.0 [5], *S*_med*j *_is the median signal on the array *j*, *S*_*TrimMeanj *_is the trimmed average on array *j *after excluding 2% of the probe sets with the highest and the lowest signals [3,4,11,22]. *NFMedian*_*j *_is obtained by using the median signal on array *j*, and *NFLogMean*_*j *_is obtained by using the mean of log transformed signals. *TS *was set to 150, 38 and 38 for *SF*, *NFMedian *and *NFLogMean*, respectively in order to have similar NFs.

In comparison with different NFs, a score, *NFscore *is introduced. *NFscore*_*j *_= (*NF*_*j *_- *TrueNF*_*j*_)/*TrueNF*_*j*_, and *TrueNF*_*j *_= (*SF*_*j *_+ *NFMedian*_*j *_+ *NFLogMean*_*j *_+ *NFTrimLogMean*_*j*_)/4, where *NFTrimLogMean*_*j*_, was calculated from equation (3) excluding the 2% of the probe sets with the highest and lowest signals, *TrueNF*_*j *_was used as a 'true' NF. Sum of 
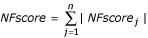
.

### Other analysis

Unless otherwise specified, logarithm transformation is carried out with the logarithm base 2. Trimmed total signal *TrimTotal *is the sum of the signals from the 2% of the probe sets with the highest signal values. Total signal *Total *is the sum of the signals of all probe sets in the array, and trimmed percentage *Tp*_*j *_= (*TrimTotal*_*j *_/ *Total*_*j*_) × 100%.

## Abbreviations

GeneChip^® ^is the registered trademark owned by Affymetrix Inc.

PM: perfect Match; MM: mismatch; SF: scaling factor; NF: normalization factor; TS: target signal Short phrase: Normalization of GeneChip microarray data

## Figures and Tables

**Table 1:** Summary of signal data in 76 rat genome U34A GeneChip microarrays.

	Lowest	Highest	Mean	SD	CV (%)
Total signal	832,561.4	3,161,392.7	2,039,655.7	526,295.0	25.80%
Sum of signals used for SF	524,513.7	1,986,236.9	1,212,296.5	336,138.0	27.73%
Trimmed total	308,047.7	1,240,257.3	827,359.1	215,325.1	26.03%
Mean signal	94.6	359.3	231.0	59.8	25.80%
Median of signals	17.8	54.8	35.7	8.7	24.41%
Mean of log signals	4.3	5.8	5.1	0.4	7.17%
Trimmed percentage	34.4	54.1	40.7	4.4	10.70%

"Total signal" is the sum of all the signals on each array. "Sum of signals used for *SF*" is the sum of signals excluding the trimmed data and used to calculate *SF*. "Trimmed total" is the sum of the 2% probe sets with the highest signals on the array. "Mean of log signals" is the mean of log_2 _transformed signals. "Trimmed percentage" = (Trimmed total/Total signal) × 100%. See also in *Methods*. The "lowest" and "highest" showed the lowest and highest number in the category among the 76 chips, respectively. The mean, standard deviation (SD) and coefficient of variation (CV) were also calculated.
